# Flexible Copper Nanowire/Polyvinylidene Fluoride Membranous Composites with a Frequency-Independent Negative Permittivity

**DOI:** 10.3390/polym15234486

**Published:** 2023-11-22

**Authors:** Kai Sun, Ao Ma, Pengtao Yang, Jinjiu Qi, Yanhua Lei, Fei Zhang, Wenxin Duan, Runhua Fan

**Affiliations:** College of Ocean Science and Engineering, Shanghai Maritime University, Shanghai 201306, China; kais@shmtu.edu.cn (K.S.); 202230410117@stu.shmtu.edu.cn (A.M.); ypt19971006@163.com (P.Y.); qijj102@163.com (J.Q.); yhlei@shmtu.edu.cn (Y.L.); zhangfei@shmtu.edu.cn (F.Z.); 17863969453@163.com (W.D.)

**Keywords:** flexible electronics, conductive polymer, low-frequency dispersion, negative permittivity

## Abstract

With the increasing popularity of wearable devices, flexible electronics with a negative permittivity property have been widely applied to wearable devices, sensors, and energy storage. In particular, a low-frequency dispersion negative permittivity in a wide frequency range can effectively contribute to the stable working performance of devices. In this work, polyvinylidene fluoride (PVDF) was selected as the flexible matrix, and copper nanowires (CuNWs) were used as the conductive functional filler to prepare a flexible CuNWs/PVDF composite film with a low-frequency dispersion negative permittivity. As the content of CuNWs increased, the conductivity of the resulting composites increased sharply and presented a metal-like behavior. Moreover, the negative permittivity consistent with the Drude model was observed when CuNWs formed a percolative network. Meanwhile, the negative permittivity exhibited a low-frequency dispersion in the whole test frequency range, and the fluctuation of the permittivity spectra was relatively small (−760 to −584) at 20 kHz–1 MHz. The results revealed that the high electron mobility of CuNWs is reasonable for the low-frequency dispersion of negative permittivity. CuNWs/PVDF composite films with a frequency-independent negative permittivity provide a new idea for the development of flexible wearable electronic devices.

## 1. Introduction

Metamaterials exhibit extraordinary physical properties, such as negative permittivity, negative magnetic permeability, and negative refractive index, which have broad application prospects in signal transmission, electromagnetic shielding, and energy storage [1,2,3]. Usually, the negative permittivity in metamaterials is realized mainly by designing artificial periodic arrays. In order to extend the large-scale production and applications of metamaterials, it is better to realize the unique properties from the intrinsic properties, such as the composition and microstructure of materials. Therefore, Fan et al. [3,4,5] proposed to use the inherent properties of materials to create and adjust the negative permittivity properties and to use randomly distributed conductive functional phases and insulating matrix materials in traditional heterogeneous composite materials to obtain negative permittivity [6,7,8]. This method of tuning the negative permittivity through microstructure and different material components opens up new ways of designing metamaterials. The generation mechanism of negative permittivity in materials is mainly derived from the polarization resonance of dipoles or the plasma oscillation of electrons. Composite materials with negative permittivity can be used in many fields, such as dielectric energy storage and pressure sensors [9,10,11]. Guo et al. [12,13] studied the electrical and mechanical properties of composites with negative permittivity. In this study, they prepared flexible composites filled with different amounts of carbon nanofibers for use in pressure sensors. Qiu et al. [14,15] developed PANI/AgNP/CF composites to tune the electromagnetic parameters by strain and effectively tune the negative permittivity and negative permeability over various microwave frequencies. However, the negative permittivity in such materials usually exhibited a strong frequency dispersion, which can cause instability of conductive electronic devices and is not conducive to impedance matching. Therefore, achieving a negative permittivity with a low-frequency dispersion is a goal for the design and application of electronic devices, which is of great significance to ensure their reliable operation [16,17,18]. Otherwise, there will be adverse effects on the performance of the device. How to achieve a negative permittivity with low-frequency dispersion is still a challenge.

It is demonstrated that electron concentration and electron mobility are the two intrinsic microscopic electrical parameters affecting the negative permittivity. When the electron concentration of the conductive filler reaches an appropriate value (i.e., the percolation threshold), the negative permittivity phenomenon appears [19,20]. Electron mobility affects the frequency dispersion, and faster electron mobility can lead to a low-frequency distribution [21,22,23]. According to the percolation theory, negative permittivity materials can be obtained by adding different contents of conductive functional phases after reaching the percolated state. Recent studies have shown that conductive nanowires such as CuNWs with a high aspect ratio can effectively reduce the percolating threshold [24,25,26], which contributes to the reduction of plasma oscillation frequency and satisfying flexibility. Due to their excellent conductivity, CuNWs can be a better candidate for functional fillers in negative permittivity materials. Moreover, CuNWs can be well bent and stretched owing to their excellent plasticity, which makes them widely used in flexible electronics, transparent conductive films, and sensors [27,28,29,30]. Compared to silver nanowires or carbon fibers, CuNWs can be more suitable for large scale production considering their lower price. On the other hand, to meet the requirements of flexibility of membranous composites, choosing an appropriate matrix material is key. PVDF powders are selected as the matrix due to their high tensile strength and strong hydrogen bonds [31,32,33].

Therefore, a flexible and stretchable PVDF was used as the matrix, and CuNWs were selected as functional fillers to fabricate CuNWs /PVDF with negative permittivity and low-frequency dispersion composite films by the tape casting method. At the same time, the composition, morphology, distribution, AC conductivity, and permittivity properties of the film at 20 kHz–1 MHz frequency were studied. When the content of CuNWs increased from 28.7 vol% to 29.6 vol%, the conductivity increased sharply, and the permittivity also changed from positive to negative. With the construction of percolative CuNWs networks, the AC conduction mechanism was transformed from hopping conduction to metal-like conduction. More importantly, the low-frequency dispersion phenomenon was observed in the composite film. When the content of CuNWs was 29.6 vol%, the negative permittivity fluctuated only from −760 to −584, and the low-frequency dispersion negative permittivity film material was successfully prepared.

## 2. Materials and Methods

### 2.1. Materials

The polyvinylidene fluoride (PVDF) was purchased from Dongguan Zhanyang Polymer Materials Co., Ltd. (Dongguan, China). CuNWs, sodium dodecylbenzene sulfonate (SDBS), and polyvinylpyrrolidone (PVP) were purchased from Sinopharm Chemical Reagent Co., Ltd. (Shanghai, China). N,N-dimethylformamide (DMF) was purchased from Shanghai Macklin Biochemical Technology Co., Ltd. (Shanghai, China). Deionized water was prepared by the laboratory.

### 2.2. Methods

**Figure 1:**
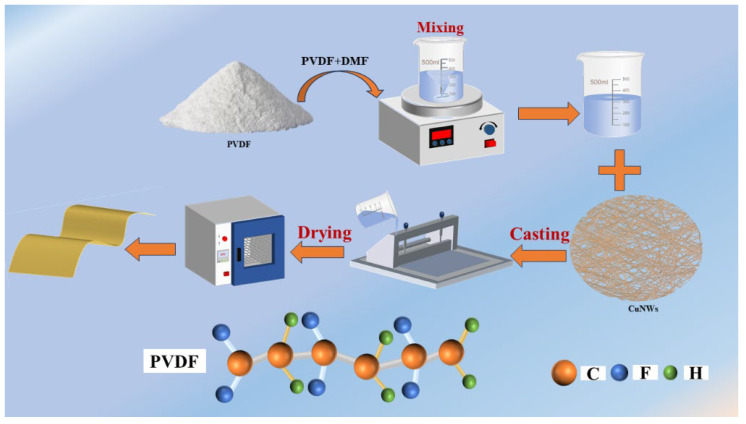
The schematic diagram of the CuNWs/PVDF membranous composites.

The material preparation process of the experiment content in this paper is the all-solid–liquid mixing assisted casting method. The overall experimental process can be described as follows. To improve the surface activity of the CuNWs, a pretreatment is necessary. The CuNWs were initially washed with deionized water and then completely dried. Afterward, sodium dodecylbenzene sulfonate and PVP powder were dissolved in deionized water, and the CuNWs were distributed by a magnetic stirrer for 0.5 h. In the end, the CuNWs were dried in a blast oven at 60 °C for 24 h. Subsequently, 0.5 g of PVDF powders were scattered in 20 mL of DMF solution and placed on a magnetic stirrer. The stirring speed was adjusted to 2000 rpm until the PVDF powder was completely dispersed and a clear and transparent mixture was obtained. The pretreated conductive functional materials were added to the PVDF mixed solution. The mixture was mechanically stirred at a speed of 1000 rpm for 0.5 h, and subjected to low power ultrasonic treatment in a nano-sonicator for 10 min to obtain a uniformly dispersed mixture. The mixture was dropped on a glass plate, and the mixture was evenly spread on the glass plate using a roll to roll coater to achieve a uniform thickness of about 400 μm. Subsequently, the coated glass plate was placed in a vacuum oven at 70 °C for 12 h. Then, the glass plate was quickly immersed in cold water to remove the film, and the successfully obtained film was dried in a convection drying oven for 12 h. The schematic diagram of the CuNWs/PVDF membranous composites is shown in Figure 1.

### 2.3. Characterization and Measurement

The microstructure of the CuNWs/PVDF samples and the distribution of CuNWs in the PVDF matrix were observed by field emission scanning electron microscopy (FESEM, Zeiss Gemini 300, Oberkochen, Germany). X-ray (XRD: (D/MaxB, Rigaku)) was used for phase analysis of the sample, and the model of the testing instrument involved was Panaco X’Pert Powder. The Cu target was used as the radiation source, the diffraction angle ranged from 10° to 90°. The Fourier transform infrared (FT-IR) spectra of samples in the range 500–4000 cm^−1^ were acquired by FT-IR spectroscopy (Thermo Scientific Nicolet IS 10, Waltham, MA, USA). The permittivity spectra of the CuNWs/PVDF composites were measured by a parallel plate method using the LCR meter (Keysight E4980AL, Los Angeles, CA, USA) from 20 kHz to 1 MHz. During the measurement, the sample was placed between two parallel metal electrodes, which were located in an external electric field. The negative permittivity can be calculated by measuring the capacitance of the capacitor and the distance between the electrodes. In order to eliminate stray capacitance at the edge of the sample, it is necessary to protect the main electrode and add protective electrodes to absorb the electric field at the edge. Gold films were sputtered on both sides of the sample to reduce measurement errors caused by air gaps. The thin film electrode is processed into a ring shape with an inner diameter of 10 mm. Then, open circuit compensation and short circuit compensation are performed to eliminate the stray admittance and residual impedance of the test fixture, respectively. After the compensation adjustment, the sample is placed in contact with the two electrodes for measurement. The resistance *R* and capacitance *C* of the sample were measured by the LCR meter at room temperature. The dielectric constant ($\varepsilon_{r}^{\prime }$) and AC conductivity ($\sigma_{ac}$) can be described as follows:

(1) $$\varepsilon_{r}^{\prime }=\frac{dC}{A\varepsilon_{0}}$$

(2) $$\sigma_{ac}=\frac{d}{RS}$$

where *d* is the sample thickness, *A* is the area of the electrode, *C* is the capacitance, $\varepsilon_{0}$ is the vacuum permittivity (8.85 × 10^−12^ F/m), *R* is the resistance, and *S* is the area of the sample.

## 3. Results and Discussion

### 3.1. Microstructural Features and Morphology

**Figure 2:**
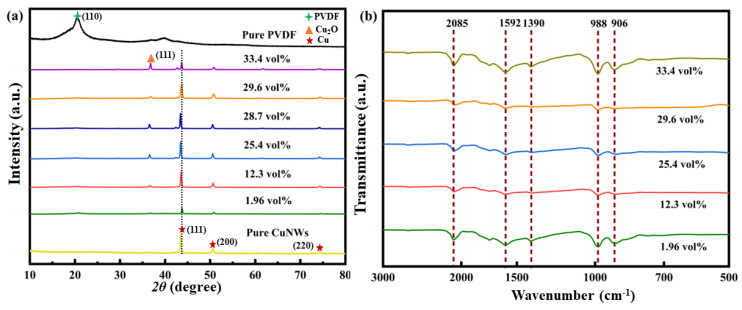
The XRD patterns (**a**) and FT-IR spectra (**b**) of CuNWs/PVDF composites with different contents of CuNWs.

The XRD patterns of pure PVDF, CuNWs, and their composites are shown in Figure 2a. The bottom curve represents the XRD pattern of pure CuNWs, and the characteristic peaks appear at 2*θ* = 43.5°, 2*θ* = 50.8°, and 2*θ* = 74.3°, corresponding to the (111), (200), and (220) crystal planes of the CuNWs. This is consistent with the face centered cubic crystal structure of CuNWs. The peak appeared at 2*θ* = 37.0° corresponding to Cu_2_O due to the oxidation of the CuNWs during the heat treatment of composites. The top curve in the graph represents the XRD pattern of pure PVDF thin film. The crystal structure of PVDF consists of alternating sequences of tetrafluoroethylene and vinylidene fluoride units, so the characteristic peaks correspond to the (110) crystal planes. The peak at circa 20.2° was related to the *β* phase.

Figure 2b presents the FT-IR spectroscopy of CuNWs/PVDF composite films. The peak at 1390 cm^−1^ corresponds to the bending vibration of C-H bonds in PVDF, which was attributed to the presence of the trifluorethylene group in the PVDF. The peak at 988 cm^−1^ is a superposition of the original characteristic peaks of fluoride in PVDF influenced by CuNWs, and it can be observed that the characteristic peaks are significantly enhanced with the increase in CuNWs in the composite. Peaks observed at 1592 cm^−1^ and 2085 cm^−1^ are new characteristic peaks generated by the interaction between CuNWs and PVDF after composite formation, and the peak intensity increases gradually with a higher proportion of CuNWs.

**Figure 3:**
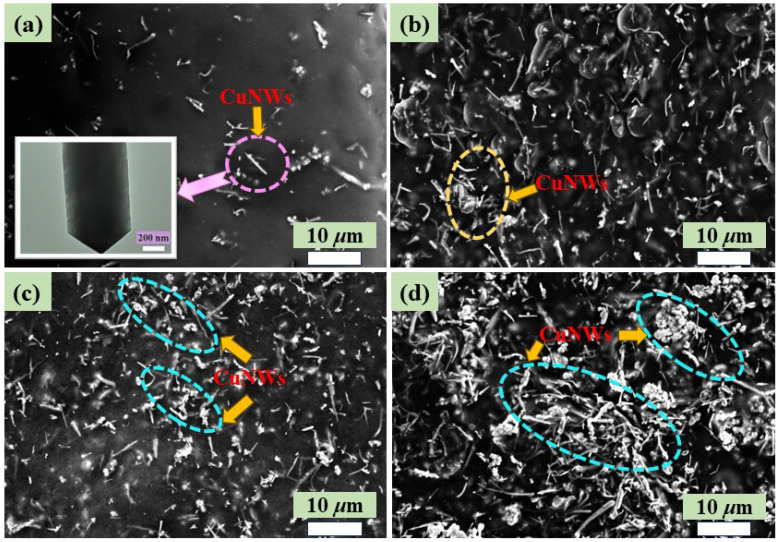
SEM images of CuNWs/PVDF composites with different CuNWs contents: (**a**) 1.96 vol%, (**b**) 12.3 vol%, (**c**) 25.4 vol%, and (**d**) 29.6 vol%.

The SEM images of CuNWs/PVDF composites shown in Figure 3a–d represent the microstructures of CuNWs/PVDF films with CuNWs contents of 1.96 vol%, 12.3 vol%, 25.4 vol%, and 29.6 vol%, respectively. The inset in Figure 3a is the TEM image of CuNWs. When the CuNWs content was 1.96 vol%, the conductive particles were scattered in the PVDF matrix and there was minimal charge flow between them. As the CuNWs content gradually increased to 12.3 vol%, the dispersion of CuNWs in the PVDF matrix became relatively uniform, with some interparticle contact and a tighter bond between CuNWs and the matrix. As the content of CuNWs increased from 12.3 vol% to 25.4 vol% in the CuNWs/PVDF composites, the spacing between the conductive particles decreased, gradually shortening until the formation of conductive pathways. From Figure 3d, it can be observed that when the concentration of CuNWs reached a higher value, they were successfully incorporated into the PVDF matrix and fused with the PVDF, resulting in a transition of the distribution of the conductive filler from initially isolated and dispersed to interconnected states, forming a dense network structure. The reason for the change in electrical properties is the transformation of microstructure and morphology. In the following analyses, special attention will be paid to the relationship between conductivity and permittivity properties, linking microscopic transitions to macroscopic property control.

### 3.2. Electrical Conductivity of Alternating Current

**Figure 4:**
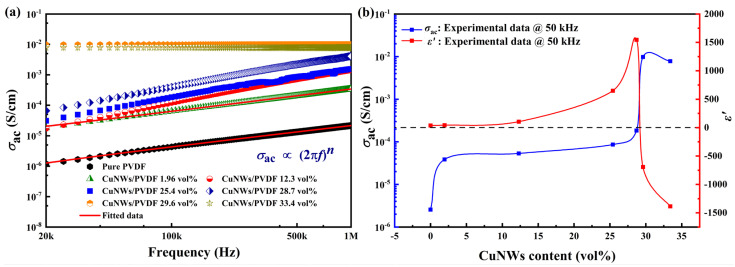
(**a**) The AC conductivity spectra. (**b**) The dependence of electrical conductivity (blue curve) and permittivity (red curve) on the content of CuNWs, respectively. The black dashed line represents the baseline of zero.

Figure 4 shows the AC electrical conductivity spectra of CuNWs/PVDF composite thin films with different volume fractions. In the whole test frequency range, the electrical conductivity of the composite thin films exhibits a positive correlation with the content of CuNWs. Moreover, for pure PVDF with a CuNWs content of 1.96 vol%, the conductivity curve follows a power law behavior: the equation *σ = σ*_ac_ *+ A(2πf)^n^* represents a power law model that describes the variation of complex conductivity with frequency for a wide range of dielectric materials, where *σ*_ac_ represents the AC electrical conductivity of the composite material, *σ* denotes the DC electrical conductivity, *A* is a constant related to the permittivity properties of the material, and *n* is a fractional exponent between 0 and 1 (0 < *n* < 1). This power law model can effectively describe the frequency dependence of complex conductivity observed in numerous experimental studies of dielectric materials. In the low-frequency regime, *σ*_ac_ is primarily determined by the DC electrical conductivity. At low CuNWs contents, the composite material exhibits an insulating state. As the CuNWs content increases from 25.4 vol% to 28.7 vol%, it falls within the percolation threshold range, and the sharp increase in conductivity confirms the occurrence of percolation behavior. However, when the CuNWs content reaches 33.4 vol%, the conductivity decreases. It was attributed to the uneven distribution of the conductive functionality within the matrix, which affects the propagation range of electrons within the matrix, leading to a reduction in conductivity.

Furthermore, for composite materials with higher volume fractions of CuNWs, the electrical conductivity remains almost unchanged with increasing frequency. This is because the conductivity of the sample mainly comes from the influence of carriers and polarization. As the content of the conductive phase increases, the DC conductivity generated by the carriers gradually exceeds the AC conductivity generated by the polarization of PVDF molecules. However, the DC conductivity generated by the carriers is almost unaffected by frequency, which results in the overall conductivity of the composite material being independent of frequency in this case. As shown in Figure 4b, the dependence of conductivity (blue curve) and dielectric constant (red curve) on CuNWs content, respectively. A significant increase in conductivity was observed when the CuNWs content reached 29.6 vol%. To validate the occurrence of electrical percolation in this range, the AC conductivity at different content levels was plotted. The red curve shows the relationship between the permittivity and content of CuNWs. The curve results indicate good agreement with the percolation theory. When the CuNWs content was relatively high, the propagation distance between electrons significantly increased, resulting in the composite material exhibiting metallic-like conductivity behavior.

### 3.3. Dielectric Properties

**Figure 5:**
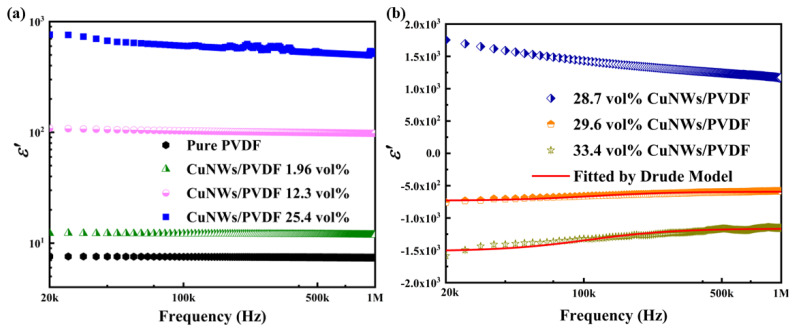
The permittivity spectra of CuNWs/PVDF composites with 0, 1.96, 12.3 and 25.4 vol% of CuNWs (**a**) and 28.7, 29.6, 33.4 vol% of CuNWs (**b**).

Figure 5 shows the real permittivity (*ε*′) for CuNWs/PVDF composites with different CuNWs contents. As shown in Figure 5a,b, *ε*′ is positive for the composites with CuNWs contents no more than 28.7 vol%. Moreover, the pure PVDF matrix exhibits relatively low permittivity, as the content of CuNWs continuously increases, the *ε*′ experiences a growth of three orders of magnitude. This is mainly due to the high interface polarization between CuNWs and the PVDF matrix, and a large amount of induced charge accumulation will occur at the interface of CuNWs and PVDF under the influence of an external AC electric field. Electrons tend to accumulate at the interface between CuNWs and PVDF, forming a charge separated region known as the Maxwell-Wagner-Sillars layer. This phenomenon is consistent with the Maxwell-Wagner-Sillers theory [34]. When the frequency of the applied electric field is low, the rate of charge separation and recombination in the Maxwell-Wagner-Sillars layer is correspondingly low, which is beneficial to the flow of charges in the composite material and increases the permittivity constant. When the content of CuNWs exceeds the percolation threshold, the CuNWs are connected, thus preliminarily forming conductive pathways. Meanwhile, with further increases in the volume fraction of CuNWs, the interconnection range among conductive fillers in PVDF gradually expands. Under the influence of plasma resonance, the real part of permittivity transitions from positive to negative. As shown in Figure 5b, the complex relationship between permittivity and frequency is exhibited with red solid lines fitted by the Drude model [35]. The measured data agree well with the calculation results. The plasma-like negative permittivity can be described by the Drude model:

(3) $$\varepsilon_{r}^{\prime }=1-\frac{\omega_{p}^{\prime }}{\omega^{2}+\omega_{\tau }^{2}}$$

(4) $$\omega_{p}=\sqrt{\frac{n_{eff}e^{2}}{m_{eff}\varepsilon_{0}}}$$

where *ω_p_* is the plasma angular frequency, *ω* is the angular frequency of the applied electric field, *ω_τ_* is the damping angular frequency, *ε*_0_ is the vacuum permittivity (8.85 × 10^−12^ F/m), *n*_eff_ is the effective concentration of conducting electrons, *m*_eff_ is the effective mass of electrons, and e is the charge of an electron (1.6 × 10^−19^ C). For composite thin films with volume fractions of CuNWs of 29.6 vol% and 33.4 vol%, the fitted *ω*_p_ values are 1.23 × 10^7^ rad/s and 8.15 × 10^6^ rad/s, respectively.

For the CuNW/PVDF composite material with 29.6 vol% of CuNWs, the negative permittivity is approximately −7.5 × 10^2^ at 20 kHz and −5.2 × 10^2^ at 1 MHz, reflecting low-frequency dispersion characteristics. Furthermore, throughout the entire testing phase, the negative permittivity remains stable at around −200, regardless of changes in frequency. This low-frequency dispersion phenomenon can be explained by Debye-type dielectric relaxation interacting with Drude-type dielectric relaxation, resulting in reduced dispersion [36].

Table 1 shows the comparison of dispersion behavior in composite materials with different negative permittivities. It can be seen from the table that some polymer based composite materials and some ceramic-based composite materials, such as graphene/polyvinyl alcohol [37], graphene/PDMS [38], and WC/polypyrrole [39], show strong negative permittivity. However, these ε′ vary greatly with frequency, exhibiting high−frequency dispersion behavior. For the curve of 29.6 vol% CuNWs, the ε′ was almost invariable with the frequency, exhibiting a low−frequency dispersive behavior.

### 3.4. Impedance Analysis

**Figure 6:**
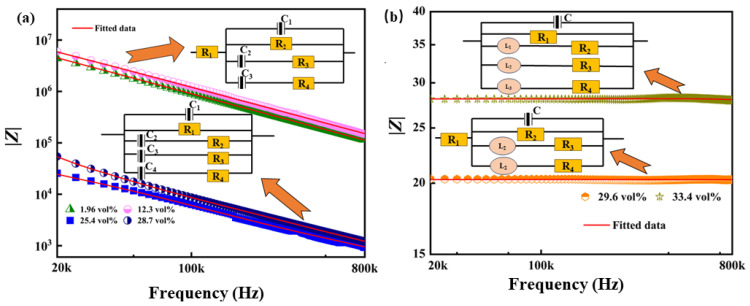
(**a**) Impedance module spectra of CuNWs/PVDF with different CuNWs contents. (**b**) The equivalent circuit.

To further elucidate the mechanism of the negative permittivity, it was possible to study it in conjunction with the impedance analysis of CuNWs/PVDF composite thin films. The fitting of equivalent circuits can effectively combine impedance with negative permittivity. Figure 6a,b illustrate the impedance spectrum and equivalent circuit diagram of CuNWs/PVDF composite thin films when the reactance values were negative. Theoretically, when a capacitor in a circuit was subjected to voltage, generating an electric field, and the direction of the current flowing due to the accumulation of charge in the capacitor was opposite to the direction of the voltage, the reactance of the capacitor exhibited a negative value. Figure 6a depicts the spectrum when the reactance values of the CuNWs/PVDF composite thin film were positive. A positive reactance occurred when the current variation in an inductor affected the electromagnetic field. The inductor stored a certain amount of energy under the influence of the electromagnetic field. If the direction of the current variation was opposite to the direction of the potential variation, the reactance of the inductor was positive. Overall, the impedance values decreased gradually with increasing CuNWs content, which indicated an overall increase in the concentration of free electrons in the composite thin film. This trend was consistent with the variations shown in the AC conductivity spectrum in Figure 4a. From the equivalent circuit diagram, it can be observed that when the CuNWs content was below 12.3 vol%, it can be fitted as a parallel combination of two capacitors, *C*_1_ and *C*_2_. As the content of the conductive phase continues to increase, the capacitance effect is enhanced, and it can be fitted as a circuit consisting of three capacitors, *C*_1_, *C*_2_, and *C*_3_, in parallel. For samples with negative permittivity, the equivalent circuit exhibited a combination of resistors, inductors, and resistors. With the increase in electron concentration, the inductance characteristics were gradually enhanced. From Figure 6b, it can be observed that when the CuNWs content increased to 33.4 vol%, the inductance elements in the circuit increased from two (*L*_1_ and *L*_2_) to three (*L*_1_, *L*_2_, and *L*_3_). The relationship between reactance and permittivity can be described by the following expression:

(5) $$|Z_{r}|=\sqrt{{\left|Z^{\prime }\right|}^{2}+{\left|Z^{\prime \prime }\right|}^{2}}$$

(6) $$\varepsilon_{r}^{\prime }=-\frac{Z^{\prime \prime }}{2f\pi c_{0}(Z^{\prime 2}+Z^{\prime \prime 2})}$$

where *f* is the testing frequency, *C*_0_ is the vacuum capacitance, *Z*′ represents the real part of the impedance, and *Z*″ represents the imaginary part of the impedance. When the permittivity is negative, the reactance value is positive; the reason is that when the composite film has a negative permittivity, the overall performance has the inductance characteristic. Therefore, the negative permittivity is a result of inductance characteristics [44].

## 4. Conclusions

The positive permittivity of CuNWs/PVDF membranous composites increased due to the enhanced interface polarization between CuNWs and PVDF powders, as the volume fraction of CuNWs increased. After the content of CuNWs reached 29.6 vol%, the permittivity of the composites changed from positive to negative values because of the formation of a connective network, and the negative permittivity spectra exhibited a low-frequency dispersion. According to equivalent circuit analysis, the negative permittivity is ascribed to the inductive characteristic, while the positive permittivity is related to the capacitive characteristic. The low-frequency dispersion of negative permittivity results from the high electron mobility of CuNWs, which contributes to hopping electrons to keep up with the variation of the external electric field. It is worth noting that the negative permittivity was observed when the content of CuNWs reached 29.6 vol%. In fact, such a high loading level is not conducive to the strength and limits practical applications. In future work, the mechanical properties including the strength, flexibility, toughness, and durability of such composites should be also considered. This study opens up an approach to flexible composites and such membranous composites with a frequency independent negative permittivity provide guidance for the design of wearable devices, sensors, and cloaking.

## Figures and Tables

**Table 1:** Comparison of dispersion behavior in composite materials with different negative permittivities.

Composites Category	Fillers Content	Dispersion (Beginning–End)	Frequency Range	References
GR/PVA	26%	−5.5 × 10^4^ to −3.0 × 10^4^	10 kHz–1 MHz	[37]
GR/PDMS	4%	−6.1 × 10^4^ to −3.0 × 10^4^	10 kHz–1 MHz	[38]
WC/PPy	60%	−3.0 × 10^3^ to −300	10 kHz–1 MHz	[39]
PVA/GR	20%	−4.8 × 10^3^ to −480	10 kHz–1 MHz	[40]
PVP@GR/PVDF	25%	−480 to −50	10 MHz–1 GHz	[41]
PVA/CB	2.5%	−450 to 0	10 kHz–10 MHz	[42]
PDMS/CCFeNP	50%	−4 × 10^3^ to −400	100 Hz–100 MHz	[43]
CuNW/PVDF	29.6%	−760 to −584	20 kHz–1 MHz	This work

## Data Availability

Data are contained within the article.
